# Correlation analysis of postoperative cognitive function and event-related potentials in patients undergoing general anesthesia

**DOI:** 10.1097/JS9.0000000000003164

**Published:** 2025-08-07

**Authors:** Xiuqin Rao, Conghui Wei, Pengcheng Yi, Chubing Long, Qiang Huang, Xifeng Wang, Maoling Zhong, Yueyang You, Fuzhou Hua, Yue Lin

**Affiliations:** a Department of Anesthesiology, The Second Affiliated Hospital, Jiangxi Medical College, Nanchang University, Nanchang, China; b Key Laboratory of Anesthesiology of Jiangxi Province, Nanchang, Jiangxi, China; c Department of Rehabilitation Medicine, The Second Affiliated Hospital of Nanchang University, Nanchang, China; d Department of Anesthesiology, The First Affiliated Hospital, Jiangxi Medical College, Nanchang University, Nanchang, China; e Department of Anesthesiology, The First Affiliated Hospital of Gannan Medical University, Ganzhou, China

**Keywords:** cognitive dysfunction, event-related potentials, general anesthesia, N200, P300, postoperative delirium

## Abstract

**Background:**

As the population ages, both the proportion of elderly surgical patients and the incidence of postoperative delirium (POD) are rising. Due to the challenges in identifying POD, misdiagnosis and missed diagnosis remain common, highlighting the need for objective cognitive assessment tools.

**Objective:**

This article aims to compare event-related potentials (ERPs) characteristics between patients with and without POD following general anesthesia, and to evaluate the potential of ERPs as predictive diagnostic markers for POD.

**Methods:**

POD was assessed in 28 patients with gastrointestinal tumors using the 3D-CAM scale on postoperative days 1, 3, and 7. Task-related electroencephalograms (EEGs) were recorded using a 64-channel system approximately 10 days after surgery. ERP components P300 and N200, along with task accuracy, were analyzed to evaluate electrophysiological changes associated with POD.

**Results:**

P300 and N200 latencies were significantly prolonged and amplitudes reduced in patients with delirium. Receiver Operating Characteristic (ROC) curve analysis revealed strong diagnostic efficacy of ERP latency parameters in identifying POD

**Conclusions:**

Patients with POD demonstrated significant alterations in ERPs, particularly prolonged P300 and N200 latencies and a trend toward reduced amplitudes. These findings suggest that P300 and N200 may serve as promising, non-invasive electrophysiological markers for the detection of delirium; however, further studies with larger sample sizes and longitudinal designs are needed to validate their diagnostic utility.

## Introduction

With the accelerating aging of the population, recent census data show that individuals aged 60 and above comprise 18.7% of the Chinese population. In 2021 alone, more than 70 million surgeries were performed in China, with over 20 million involving elderly or critically ill patients – a growing proportion of all surgical cases. Perioperative neurocognitive disorders (PNDs) encompass a range of cognitive impairments occurring before, during, or after surgery, commonly manifesting as memory loss, impaired concentration, or global cognitive decline. PND includes several subtypes, such as preoperative cognitive impairment, postoperative delirium (POD), and postoperative cognitive dysfunction (POCD). POD is a common perioperative complication involving central nervous system dysfunction, particularly among critically ill and elderly patients. It is primarily characterized by attention deficits, disorientation, cognitive disturbances, and impairments in learning and memory functions^[1]^.

**HIGHLIGHTS**

- This is the first study to investigate the association between postoperative delirium (POD) and the P300 and N200 components of event-related potentials (ERPs).
- P300 and N200 latencies are significantly prolonged in delirium patients at multiple midline electrodes (e.g., CPz, Pz, and Cz), indicating impaired cognitive processing speed and attention allocation.
- ERP latency measures demonstrate high diagnostic efficacy for POD, with AUC values >0.7 and the N200 latency at POz reaching an AUC of 0.988.
- Patients with POD show significantly lower task accuracy and longer reaction times, consistent with ERP findings.
- The integration of 64-channel high-density electroencephalogram, a standardized oddball paradigm, and behavioral data provides a millisecond-level, objective framework for assessing postoperative cognitive function.

Numerous clinical studies have identified multiple risk factors for postoperative cognitive decline, with advanced age and surgical intervention being well-established contributors^[2]^. Reports indicate that POD occurs in 11%–51% of patients undergoing major surgery, and the incidence may rise to 50%–70% in high-risk populations^[3,4]^. According to the Chinese Expert Consensus on the Prevention and Treatment of Postoperative Delirium in Elderly Patients, the overall incidence in individuals over 65 years old is 12.0%, with a reported rate of 18.1% following upper abdominal surgery.

Moreover, delirium is not always a transient condition. A meta-analysis of 23 studies has demonstrated a strong association between delirium in hospitalized patients and an increased risk of long-term cognitive decline^[5]^. In some cases, subtle structural brain damage during delirium may result in irreversible cognitive impairment. Studies have shown that only 4% of elderly patients fully recover after discharge, while up to 80% continue to experience residual cognitive deficits six months later^[6]^. Patients with POD have a significantly higher likelihood of developing POCD compared to those without delirium^[7]^. POD also leads to prolonged hospitalization, increased medical costs, reduced functional capacity, and substantially elevated risks of long-term cognitive impairment, dementia, and mortality^[8,9]^. However, current assessment tools rely heavily on subjective evaluation, contributing to frequent misdiagnosis and missed diagnosis. There is a pressing need for objective and reliable criteria to assess POCD.

Electroencephalogram (EEG) is a non-invasive technique that records real-time electrical activity in the brain. It has proven valuable in distinguishing delirium from other neurological disorders^[10]^. In delirious patients, EEG often reveals generalized slowing of background activity, which may reflect underlying neurophysiological disruptions. Event-related potentials (ERPs) are voltage fluctuations in the brain elicited by specific sensory or cognitive stimuli, typically occurring at stimulus onset or offset^[11]^. Through time-locked averaging and signal processing, distinct ERP components associated with cognitive processing can be isolated. Because endogenous ERPs are closely linked to cognitive functions, they are often referred to as cognitive potentials^[12]^. As objective indicators of cognitive processing, ERPs are widely used in studies of visual perception, attention, language, and memory, offering direct insights into brain electrophysiological activity.

ERPs may serve as biomarkers to aid in the early identification of neuropsychiatric disorders and the exploration of disease progression^[13]^. Thanks to their ability to directly capture neural electrical activity with millisecond precision, ERPs enable real-time assessment of cognitive processing, making them a powerful and non-invasive tool for objectively evaluating brain function^[14]^. In cognitive neuroscience, ERPs are often employed in lesion studies to link behavioral impairments with disrupted neuronal processing, using their high temporal resolution to probe attention and memory mechanisms at the millisecond scale^[15]^. The millisecond-level temporal resolution of ERPs allows precise tracking of neural events during cognitive tasks, making them a powerful tool for exploring brain function^[16]^.

The P300 component, typically observed between 250 and 800 ms post-stimulus, is associated with conflict monitoring and inhibitory control. The P300 component is generally divided into two subtypes: P3a and P3b^[17]^. P3a and P3b are generated by distinct cortical systems: P3a arises primarily from frontal regions and is involved in bottom-up attentional orienting and novelty detection, while P3b originates in parieto-occipital areas and reflects higher-order processes related to memory and top-down attention^[18]^. The P300 reflects coordinated neural activity across the frontal, parietal, temporal, and occipital lobes, representing the brain’s effort to detect and differentiate task-relevant stimuli^[18]^. Unlike early ERP components such as P100 or N100, the P300 engages widespread bilateral cortical regions, consistent with its involvement in diverse cognitive functions^[19]^. Reductions in P300 amplitude are correlated with cognitive impairment, making it a potential biomarker for cognitive vulnerability^[20]^, As noted, the N200 (200–350 ms) is linked to conflict detection and inhibitory control^[21]^. Like the P300, it is involved in attentional, mnemonic, and executive processes^[22]^. In memory research, the N200 is associated with encoding, storage, and retrieval processes in both working and long-term memory. Alterations in N200 amplitude may reflect changes in memory familiarity and recognition^[23]^. Both P300 and N200 are highly sensitive to anesthetic and surgical effects, aligning with the attentional and executive deficits seen in POD. Their changes offer mechanistic insights that extend beyond what behavioral assessments can capture.

This study aimed to assess the relationship between neural activity and cognitive function by directly recording EEG signals. ERP characteristics may serve as objective indicators for evaluating postoperative cognitive impairment.

This cohort study has been reported in line with the STROCSS guidelines^[24]^.

## Methods

### Study design

This was a prospective observational cohort study.

### Sample size calculation

Based on existing literature, the estimated incidence of short-term cognitive dysfunction after delirium is 89%, compared to 37% in non-delirium patients^[7]^. With a Type I error (α) of 0.05 and a Type II error (β) of 0.1, the calculated minimum sample size was 9 participants in the delirium group and 18 in the non-delirium group, for a total of 27 participants.

### Anesthetic protocol

All patients underwent surgery under general anesthesia. Induction was performed using 2 mg/kg propofol, 1 μg/kg sufentanil, 0.05–0.1 mg/kg midazolam, and 1 mg/kg rocuronium. Anesthesia was maintained with 1–1.5 MAC sevoflurane and remifentanil infusion (0.1–0.2 μg/kg/min), along with continuous propofol infusion at 1 mg/kg/h. Bispectral Index (BIS) values were maintained between 40 and 60. Volume-controlled ventilation was adjusted to maintain end-tidal CO_2_ between 35 and 45 mmHg. At the end of surgery, 1 mg atropine and 4 mg neostigmine were administered to reverse muscle relaxation.

### Study participants

Patients who underwent gastrointestinal tumor resection at the East Lake Campus of the Second Affiliated Hospital of Nanchang University between May and December 2023 were enrolled. Only those with normal preoperative cognitive function, confirmed by the Mini-Mental State Examination (MMSE), were included. Delirium was assessed postoperatively using the 3D-CAM on postoperative days 1, 3, and 7. Based on these evaluations, patients were categorized into delirium and non-delirium groups. The final dataset included 9 patients with delirium and 19 without.

### EEG data acquisition

**Figure 1.**
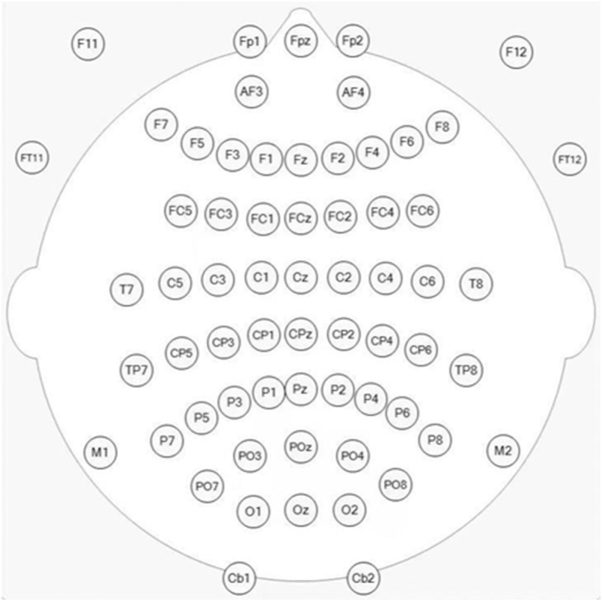
Sixty-four electrodes according to the international 10–20 system.

EEG recordings were scheduled for approximately the 10th postoperative day. If patients were discharged earlier, EEG was performed prior to discharge. If complications delayed EEG beyond day 10, data were collected as soon as possible thereafter. EEG signals were recorded using the Neuroscan system and analyzed using Scan 4.5 software. A 64-channel EEG cap (Ag/AgCl electrodes) (Figure 1) was used in accordance with the international 10–20 system. Signals were amplified via the SynAmps2 amplifier, with a maximum sampling rate of 20 000 Hz. Filters were set at 0.05–3500 Hz. Impedance was kept below 5 Ω.

**Figure 2.**
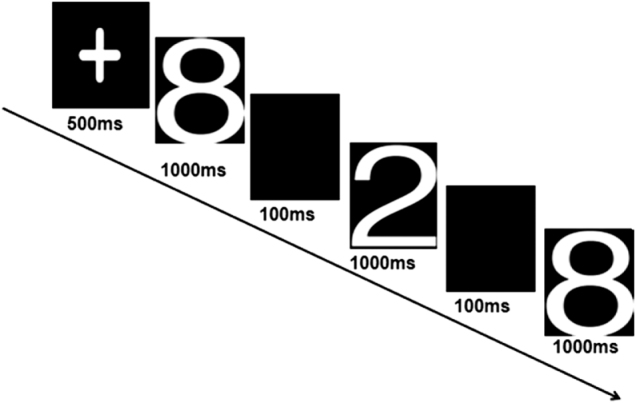
A standard oddball paradigm.

### Experimental paradigm

A standard visual oddball paradigm was designed using E-Prime software. The stimuli consisted of the numbers “2” (target) and “8” (standard). The target appeared 80 times (10%), and the standard 720 times (90%), in random order. Each stimulus lasted 50 ms with a 1-s interstimulus interval. Participants were instructed to press the left mouse button as quickly as possible upon seeing the target number “2” and to ignore the standard stimulus (Figure 2).

### EEG recording procedure

EEG acquisition was conducted at the patient’s bedside. After confirming patient readiness and explaining the procedure, the EEG cap was placed, and conductive gel was applied. Patients were instructed to remain awake, minimize movement and blinking, and sit quietly. First, a 5-min resting-state EEG was recorded. Then, participants performed the oddball task, while EEG data were continuously recorded for approximately 6 min and 30 s.

### EEG data processing

EEG data were processed offline using Scan 4.5. Data were recorded in AC mode at 1000 Hz, with a bandpass filter of 1–30 Hz. Eye-blink artifacts were detected and removed using Vertical Electro-Oculography Electrode (VEOG) channels and E-Prime synchronization. Signals were segmented and baseline corrected. Artifacts were excluded automatically. ERP components were obtained by averaging across trials.

### Clinical data collection

#### Preoperative data

Baseline variables included demographic information, medical history, vital signs, body mass index (BMI), age, education level, MMSE score, American Society of Anesthesiologists (ASA) Physical Status Classification , comorbidities, smoking and alcohol use, and preoperative hemoglobin and albumin levels.

#### Intraoperative data

All patients received standard monitoring (electrocardiogram, invasive BP, and pulse oximetry). Data collected included intraoperative blood pressure, glucose levels, surgery duration, and medication dosages (e.g., midazolam and atropine). BIS was kept between 40 and 60 throughout the operation.

#### Postoperative data

Delirium was assessed on postoperative days 1, 3, and 7 using the 3D-CAM^[25]^, pain scores (Numeric Rating Scale, NRS), and levels of hemoglobin, albumin, and CRP were recorded on the same days. Adverse events such as nausea, vomiting, dizziness, and headache were also documented. Before discharge, both resting-state and task-state EEGs were collected.

### Statistical analysis

Statistical analyses were conducted using SPSS. Continuous variables were expressed as mean ± standard deviation (X ± S) and compared using independent-sample *t*-tests or Mann–Whitney *U* tests. Categorical data were analyzed using chi-squared or Fisher’s exact test. ROC curve analysis was used to evaluate diagnostic performance.

Primary outcomes, such as ERP latency and amplitude, were analyzed using independent-sample *t*-tests, with False Discovery Rate (FDR) correction for multiple comparisons. Secondary outcomes, including biochemical markers, intraoperative vital signs, and postoperative adverse reactions, were analyzed using the Mann–Whitney *U* test for non-normally distributed data. Pearson or Spearman correlation tests were applied as appropriate.

## Results

### EEG findings

#### Comparison of P300 latency and amplitude between delirium and non-delirium groups

**Figure 3.**
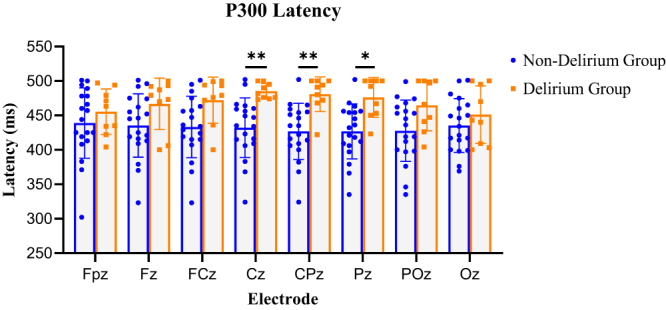
Comparison of P300 latency between non-delirium and delirium groups. Selecting the nine electrodes with the most significant amplitude, including Fpz, Fz, FCz, Cz, CPz, Pz, POz, and Oz for P300 latency statistical analysis. Using a *t*-test to compare latency between delirium and non-delirium patients. The delirium group exhibits significantly increased latency, with statistically significant differences at CPz, Pz, and POz. (Orange indicates delirium, and blue indicates non-delirium. *P < 0.05; **P < 0.01; ***P < 0.001.)

Given that ERP components typically exhibit maximal amplitude at midline central-parietal sites, midline electrodes were selected for analysis. As shown in Table 1, P300 latency was significantly prolonged in the delirium group at Cz, CPz, and Pz electrodes (*P* < 0.05, FDR-corrected). Visual inspection further confirmed that P300 latency was generally prolonged across most electrodes in the delirium group, with significant differences observed at C2, CP2, Pz, CPz, Cz, C1, C4, P2, PO3, CP1, P4, C3, P1, P8, P6, CP3, CP6, PO4, FC1, O2, CP5, PO8, POz, and CP4 (*P* < 0.05) (Fig. 3). These sites are primarily located in the central and right parietal-occipital regions.

**Table 1:** Comparison of P300 latency between non-delirium and delirium groups

Electrode	P300 latency (ms)		*P*	P-FDR*
	Non-delirium group	Delirium group		
Fpz	439 ± 51	455 ± 33	0.475000	0.475000
Fz	435 ± 46	467 ± 46	0.121000	0.162000
FCz	433 ± 45	472 ± 45	0.028600	0.057100
Cz	432 ± 43	485 ± 43	0.000626	**0.004240**
CPz	427 ± 41	481 ± 41	0.001060	**0.004240**
Pz	427 ± 40	476 ± 40	0.007800	**0.020800**
POz	428 ± 45	465 ± 45	0.048900	0.078300
Oz	435 ± 39	451 ± 39	0.445000	0.475000

*The resulting p-FDR were adjusted for multiple testing using the FDR method, yielding FDR-adjusted p-values

**Figure 4.**
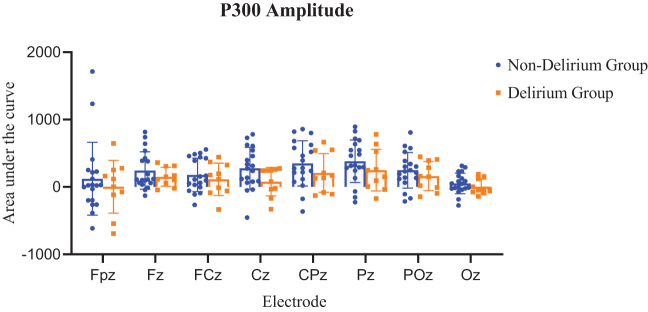
Comparison of P300 amplitude between non-delirium and delirium groups. Selecting the nine electrodes with the most significant amplitude, including Fpz, Fz, FCz, Cz, CPz, Pz, POz, and Oz for P300 amplitude statistical analysis. Using a *t*-test to compare latency between delirium and non-delirium patients. The amplitudes in the delirium group exhibited a downward trend; however, the difference was not statistically significant. Orange indicates delirium, and blue indicates non-delirium.

Amplitude was quantified as the area under the curve within a 100-ms window centered on the latency peak. While P300 amplitude appeared lower in the delirium group across most electrodes, these differences did not reach statistical significance (*P* > 0.05) (Fig. 4).

#### Comparison of N200 latency and amplitude between groups

**Figure 5.**
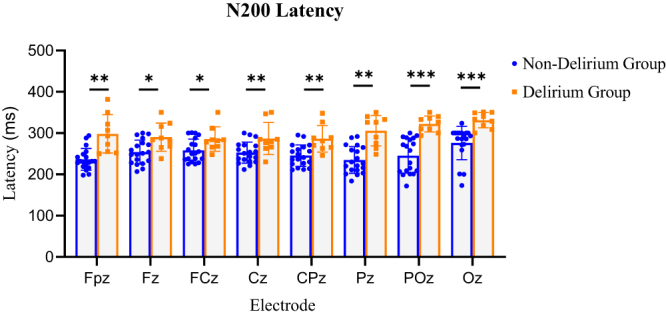
Comparison of N200 latency between non-delirium and delirium groups. Selecting the nine electrodes with the most significant amplitude, including Fpz, Fz, FCz, Cz, CPz, Pz, POz, and Oz for N200 latency statistical analysis. Using a *t*-test to compare latency between delirium and non-delirium patients. The latency of N200 in the delirium group was significantly prolonged, achieving statistical significance. (Orange indicates delirium, and blue indicates non-delirium. *P < 0.05; **P < 0.01; ***P < 0.001).

N200 latency was significantly prolonged in the delirium group at all analyzed midline electrodes: Fpz, Fz, FCz, Cz, CPz, Pz, POz, and Oz (*P* < 0.05, FDR-corrected; Table 2). Visual inspection of individual EEG traces showed widespread N200 latency prolongation in the delirium group, with statistically significant differences at numerous electrodes, including PO3, PO7, POz, P1, P3, P7, Pz, PO8, CP6, O2, Oz, Fp1, P5, P2, O1, AF3, P6, PO4, F3, FC5, CP1, F5, Fpz, C4, CP2, F7, TP8, F8, F6, FT12, P8, F4, FC4, CP4, CP3, C6, FC6, and M1 (*P* < 0.05) (Fig. 5). These electrodes were mainly located in the bilateral occipital, central-parietal, and left frontal regions.

**Table 2:** Comparison of N200 latency between non-delirium and delirium groups

Electrode	N200 latency (ms)		*P*	P-FDR*
	Non-delirium group	Delirium group		
Fpz	236 ± 26	298 ± 47	0.0005190	0.0011500
Fz	254 ± 29	290 ± 34	0.0194000	0.0259000
FCz	258 ± 28	286 ± 30	0.0489000	0.0489000
Cz	253 ± 25	287 ± 39	0.0252000	0.0288000
CPz	245 ± 26	286 ± 32	0.0058700	0.0093900
Pz	235 ± 32	306 ± 37	0.0005730	0.0011500
POz	245 ± 42	322 ± 19	0.0000439	0.0003520
Oz	276 ± 41	331 ± 19	0.0000933	0.0003730

*The resulting p-FDR were adjusted for multiple testing using the FDR method, yielding FDR-adjusted p-values

**Figure 6.**
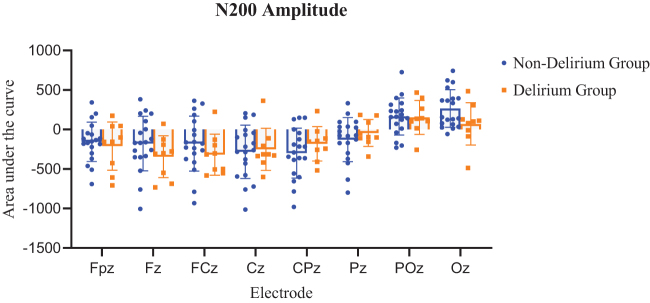
Comparison of N200 amplitude between non-delirium and delirium groups. Selecting the nine electrodes with the most significant amplitude, including Fpz, Fz, FCz, Cz, CPz, Pz, POz, and Oz for N200 amplitude statistical analysis. Using a *t*-test to compare latency between delirium and non-delirium patients. The amplitudes in the delirium group exhibited a downward trend; however, the difference was not statistically significant. Orange indicates delirium, and blue indicates non-delirium.

Similarly, N200 amplitude at CPz, Pz, POz, and Oz electrodes was lower in the delirium group, but the differences were not statistically significant (Fig. 6).

#### Superposition-averaged EEG

**Figure 7.**
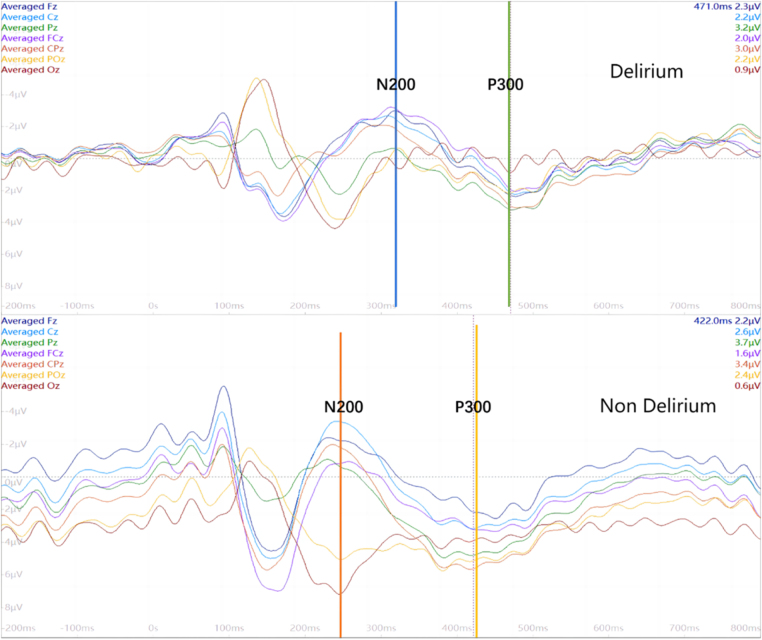
Superposition-averaged EEG. Offline data analysis was conducted using Neuroscan 4.5 software. E-Prime software was used to remove noise and eye movement artifacts, integrate EEG data, and then separately superimpose and average the EEG data for both groups. By visually comparing the latency differences of N200 and P300 between the delirium group and the non-delirium group, it was shown that the delirium group had significantly delayed latencies and reduced amplitudes.

Superposition-averaged EEG waveforms were computed for both groups at midline electrodes (Fz, FCz, Cz, CPz, Pz, POz, and Oz). As illustrated in Figure 7, the delirium group exhibited clearly delayed N200 and P300 latencies and reduced amplitudes compared to the non-delirium group.

#### Behavioral performance in task-state EEG

**Figure 8.**
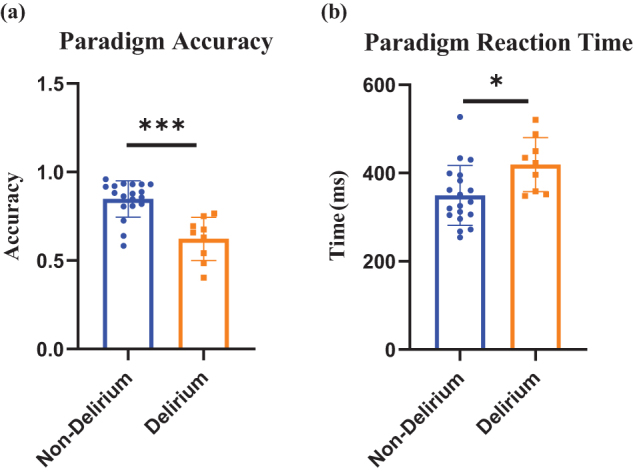
Comparison of paradigm accuracy and reaction time between non-delirium and delirium groups. (a) Paradigm accuracy. (b) Paradigm reaction time. Extract the accuracy rate and reaction time of the paradigm from the E-Prime software, and use a *t*-test for statistical analysis between the delirium group and the non-delirium group, concluding that the delirium group has a lower paradigm accuracy rate and longer reaction time. (Orange indicates delirium, and blue indicates non-delirium. *P < 0.05; **P < 0.01; ***P < 0.001).

Behavioral data from the oddball task were analyzed for accuracy and reaction time. As shown in Figure 8, patients in the delirium group had significantly lower accuracy and longer reaction times compared to the non-delirium group (*P* < 0.05). These results are consistent with the ERP findings.

#### Diagnostic performance of ERP latency measures

**Figure 9.**
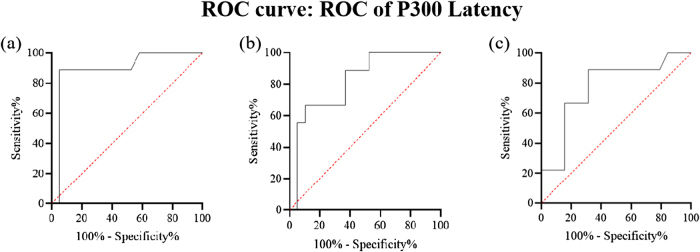
ROC curve of P300 latency for diagnosing POD. (a) ROC curve: ROC of P300 latency – CP_Z_. (b) ROC curve: ROC of P300 latency – Pz. (c) ROC curve: ROC of N200 latency – Cz.

Based on the ERP results, electrodes with the most significant group differences were selected for ROC analysis. For P300 latency, CPz, Pz, and Cz were chosen. The AUC values were 0.8918, 0.8187, and 0.7836, respectively, indicating good diagnostic performance with high sensitivity and moderate specificity (Table 3, Figure 9).

**Table 3:** P300 latency for diagnosing POD

Electrode	AUC	Sensitivity	Specificity
CPz	0.8918	88.89%	68.42%
Pz	0.8187	88.89%	63.36%
Cz	0.7836	88.89%	52.63%

**Figure 10.**
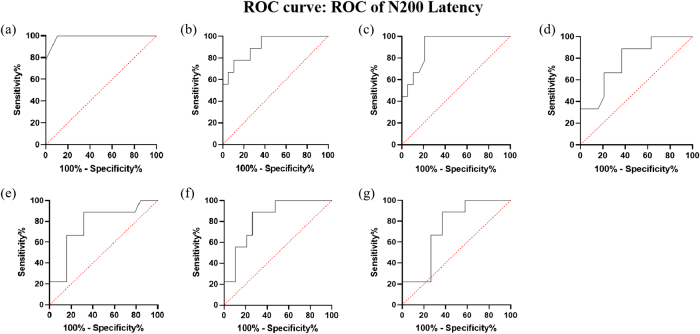
ROC curve of N200 latency for diagnosing POD. (a) ROC curve: ROC of N200 latency – POz. (b) ROC curve: ROC of N200 latency – FPz. (c) ROC curve: ROC of N200 latency – Pz. (d) ROC curve: ROC of N200 latency – CPz. (e) ROC curve: ROC of N200 latency – Fz. (f) ROC curve: ROC of N200 latency – Cz. (g) ROC curve: ROC of N200 latency – FCz.

For N200 latency, seven electrodes were analyzed: POz, Fpz, Pz, CPz, Fz, Cz, and FCz. Notably, N200 latency at the POz electrode showed excellent diagnostic value, with an AUC of 0.9883, 100% sensitivity, and 89.47% specificity. Other electrodes also showed strong diagnostic utility (Table 4, Figure 10).

**Table 4:** N200 latency for diagnosing POD

Electrode	AUC	Sensitivity	Specificity
POz	0.9883	100%	89.47%
FPz	0.9152	100%	78.95%
Pz	0.9123	100%	63.16%
CPz	0.8304	88.89%	73.68%
Fz	0.7807	88.89%	63.16%
Cz	0.7690	88.89%	68.42%
FCz	0.7368	88.89%	63.16%

### Clinical characteristics

#### Demographics

A total of 28 male patients were included: 9 in the delirium group and 19 in the non-delirium group. As shown in Table 5, there were no significant differences between the two groups in age, BMI, residence, education level, ASA classification, comorbidities, or lifestyle factors (*P* > 0.05).

**Table 5:** Comparison of general information between non-delirium and delirium groups

	Non-delirium group	Delirium group	*P*
Age (years)	71.1 ± 4.70	70.9 ± 4.98	0.855453
BMI	20.75% ± 3.28%	20.31% ± 3.75%	0.663830
Residence			0.704348
Urban	8 (42.11%)	6 (66.67%)	
Rural	11 (57.89%)	3 (33.33%)	
Education			0.362750
Elementary school	2 (11.76%)	8 (88.89%)	
Middle school	15 (78.95%)	1 (11.11%)	
University	2 (10.53%)	0 (0.00%)	
ASA classification			0.371481
II	13 (68.42%)	8 (88.89%)	
III	6 (31.58%)	1 (11.11%)	
Underlying diseases			0.670027
Yes	7 (36.84%)	2 (22.22%)	
No	12 (63.16%)	7 (77.78%)	
Smoking and drinking habits			>0.999999
Yes	13 (68.42%)	6 (66.67%)	
No	6 (31.58%)	3 (33.33%)	

#### Intraoperative data

As shown in Table 6, surgery duration was significantly longer in the delirium group than in the non-delirium group (*P* = 0.0244). Other intraoperative parameters, such as blood pressure and glucose variability, as well as dosages of midazolam and atropine, showed no significant differences (*P* > 0.05).

**Table 6:** Comparison of surgical data between non-delirium and delirium groups

	Mean of non-delirium	Mean of delirium	*P*
Blood glucose variability	1.563	1.689	0.7758
Systolic blood pressure variability	40.47	51.11	0.0603
Diastolic blood pressure variability	18.21	21.33	0.3472
Surgery duration	3.626	4.814	0.0244
Midazolam	1.316	0.8887	0.2918
Atropine	0.8684	0.9444	0.3785

#### Postoperative complications and laboratory parameters

Postoperative complications, including pain scores and adverse symptoms (e.g., nausea, vomiting, and dizziness), did not significantly differ between groups (Table 7).

**Table 7:** Comparison of postoperative complications between non-delirium and delirium groups

	Mean of non-delirium	Mean of delirium	*P*
Pain on day 1	4.000	4.222	0.8015
Pain on day 3	3.526	3.667	0.8395
Pain on day 7	2.000	2.889	0.0691
Nausea and vomiting			>0.9999
Yes	2 (10.53%)	1 (11.11%)	
No	17 (89.47%)	8 (88.89%)	
Dizziness and headache			>0.9999
Yes	3 (15.79%)	1 (11.11%)	
No	16 (84.21%)	8 (88.89%)	

However, as shown in Table 8, preoperative hemoglobin was significantly higher in the delirium group (*P* = 0.0236), while postoperative albumin levels on days 3 and 7 were significantly lower (*P* = 0.0023 and *P* = 0.0440, respectively). Other biochemical indicators, such as postoperative hemoglobin and CRP levels, showed no statistically significant differences.

**Table 8:** Comparison of preoperative and postoperative biochemical indicators between non-delirium and delirium groups

	Mean of non-delirium	Mean of delirium	*P*
Preoperative albumin	39.54	40.52	0.5324
Preoperative hemoglobin	118.6	139.7	0.0236
Postoperative hemoglobin on day 1	118.1	120.0	0.7724
Postoperative hemoglobin on day 3	107.3	107.1	0.9375
Postoperative hemoglobin on day 7	107.5	106.7	0.9092
Postoperative CRP on day 1	159.3	122.3	0.1023
Postoperative CRP on day 3	132.1	118.3	0.6047
Postoperative CRP on day 7	61.70	78.82	0.4917
Postoperative albumin on day 1	30.80	28.73	0.1607
Postoperative albumin on day 3	33.85	30.57	0.0023
Postoperative albumin on day 7	34.26	30.63	0.0440

#### Correlation analyses

**Figure11.**
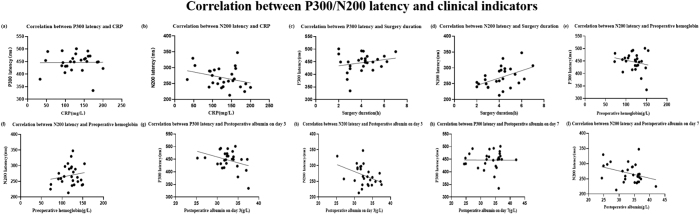
Correlation between P300/N200 latency and clinical indicators. Using Pearson’s analysis to examine the correlation between the latency of P300 and N200 with postoperative CRP, surgery duration, preoperative hemoglobin, and postoperative albumin.

Pearson’s correlation analyses were conducted to examine associations between ERP latency and clinical indicators. As shown in Table 9 and Figure 11, N200 latency was positively correlated with surgery duration (*r* = 0.4732, *P* = 0.0127); N200 latency was negatively correlated with postoperative albumin on day 3 (*r* = −0.3908, *P* = 0.0438). No significant correlations were observed between ERP latency and CRP, preoperative hemoglobin, or albumin on day 7.

**Table 9:** Correlation between P300/N200 latency and clinical indicators

	*r*	*R*^2^	95% CI	*P*
Correlation between P300 latency and CRP	0.01137	0.0001293	(−0.3702, 0.3897)	0.9551
Correlation between N200 latency and CRP	−0.3072	0.09439	(−0.6154, 0.08240)	0.119
Correlation between P300 latency and surgery duration	0.2228	0.04963	(−0.1718, 0.5557)	0.264
Correlation between N200 latency and surgery duration	0.4732	0.2239	(0.1136, 0.7232)	0.0127
Correlation between P300 latency and preoperative hemoglobin	−0.2096	0.04395	(−0.5461, 0.1851)	0.294
Correlation between N200 latency and preoperative hemoglobin	0.172	0.02959	(−0.2225, 0.5182)	0.3909
Correlation between P300 latency and postoperative albumin on day 3	−0.3548	0.1259	(−0.6475, 0.02917)	0.0694
Correlation between N200 latency and postoperative albumin on day 3	−0.3908	0.1527	(−0.6712, − 0.01269)	0.0438
Correlation between P300 latency and postoperative albumin on day 7	−0.007114	0.00005061	(−0.3861, 0.3739)	0.9719
Correlation between N200 latency and postoperative albumin on day 7	−0.3332	0.111	(−0.6331, 0.05361)	0.0894

## Discussion

POD typically presents as an acute condition characterized by fluctuating consciousness, impaired attention, disorganized thinking, and cognitive decline^[26]^. It is particularly common in elderly patients, especially those with underlying comorbidities^[3]^. POD has been shown to adversely affect both short- and long-term outcomes, including higher rates of complications, prolonged hospitalization, increased healthcare costs, and elevated perioperative mortality^[27]^. Despite its clinical significance, the diagnosis of POD largely relies on bedside evaluations using subjective scales, which can be affected by assessor experience and patient cooperation.

The 3D-CAM scale is widely used due to its high sensitivity, specificity, and ease of use^[25]^. However, challenges remain in detecting hypoactive or subclinical forms of delirium, which are often underrecognized by medical staff and family members. In some cases, subtle structural brain injury during delirium may persist beyond clinical resolution, resulting in long-term cognitive impairment and increased mortality^[6,9]^. Therefore, there is an urgent need to identify objective markers for POD that can improve detection accuracy, particularly in non-hyperactive and subclinical cases.

EEG offers an objective method for assessing brain function by capturing electrophysiological activity during cognitive processing tasks^[28]^. ERPs have been extensively applied in studies of aging, mild cognitive impairment, and dementia^[11,29]^. ERP parameters, particularly latency and amplitude, reflect the speed and intensity of cognitive processing, with delayed latency indicating slower neural transmission or insufficient cognitive resource allocation^[12,30]^. Specifically, P300 latency is thought to reflect the time required for cortical processing of cognitive tasks, while its amplitude is closely associated with memory maintenance and retrieval capacity^[31,32]^.

ERP abnormalities, including prolonged P300 and N200 latencies and reduced amplitudes, have been observed in a range of cognitive disorders and somatic diseases with central nervous system involvement^[33,34]^. These neurophysiological changes may not always correspond to traditional neuropsychological test results, as ERPs are often more sensitive to early or subtle deficits^[35,36]^. Furthermore, ERP has been widely used to evaluate cognitive changes in conditions such as cerebral ischemia, with improved ERP parameters reflecting recovery of cognitive function^[37,38]^. And ERP method is more sensitive than standard cognitive testing. Collectively, these findings support the utility of ERPs as diagnostic tools for identifying and tracking cognitive dysfunction.

In the present study, we used high-density EEG to investigate changes in ERP components P300 and N200 in patients with and without POD following general anesthesia. Compared with the non-delirium group, patients with POD exhibited significantly prolonged P300 and N200 latencies and a trend toward reduced amplitudes, consistent with prior ERP studies in neurocognitive disorders. These findings suggest persistent deficits in cognitive processing speed and attentional resource allocation in the delirium group, even after resolution of overt symptoms.

Amplitude was quantified using the area under the curve within a 50-ms window centered on peak latency. Although amplitude appeared lower in the delirium group, differences did not reach statistical significance. Several explanations are possible: (1) the window width may have been too narrow or wide, masking real amplitude differences; (2) the limited sample size may have reduced statistical power; and (3) delirium may primarily affect processing speed rather than neural firing intensity. Further validation in larger cohorts and with refined analysis techniques is warranted.

Using ERPs to detect early signs of cognitive dysfunction could be invaluable, as they can identify neural cognitive damage before behaviorally observable symptoms appear. Additionally, ERPs can pinpoint abnormalities in processing stages that behavioral measurements cannot reach^[37]^. Importantly, our results showed that latency differences were most pronounced at midline parietal electrodes, particularly CPz, Pz, and Cz for P300, and POz, Fpz, Pz, CPz, Fz, Cz, and FCz for N200. ROC analysis demonstrated that these ERP parameters had strong diagnostic utility, with AUC values >0.7 and high sensitivity/specificity, particularly for N200 latency at POz (AUC = 0.988). These findings support the use of ERP latency, especially N200, as a non-invasive and objective biomarker for early detection of POD.

The use of 64-channel EEG, while technically demanding, revealed that ERP alterations in POD are primarily localized to the occipitoparietal regions. This observation suggests that future development of portable EEG systems focusing on these areas could improve the clinical feasibility of ERP-based POD screening tools.

Among perioperative variables, only surgery duration showed a significant difference between groups, with longer operations associated with a higher risk of POD. This finding is consistent with the theory that prolonged exposure to anesthesia or intraoperative stress may exacerbate neuronal vulnerability^[39,40]^. Systolic blood pressure variability also showed a trend toward significance, potentially implicating intraoperative cerebral hypoperfusion as a contributing factor. Other factors – including age, BMI, ASA classification, comorbidities, medication use, and postoperative complications – did not significantly differ between groups, possibly due to the relatively homogeneous study population and standardized anesthesia protocol.

Notably, postoperative albumin levels on days 3 and 7 were significantly lower in the delirium group. Hypoalbuminemia has been identified as a predictor of delirium in prior studies^[41]^. Low albumin may impair inflammatory regulation, reduce antioxidant capacity, and hinder neurotransmitter synthesis. Inflammatory cytokines such as IL-1β and TNF-α, normally bound by albumin, may become elevated in hypoalbuminemic states, triggering microglial activation and hippocampal damage^[42]^. Additionally, reduced albumin thiol groups may elevate oxidative stress, promoting neuronal apoptosis.

Interestingly, our results showed that preoperative hemoglobin levels were higher in the delirium group, contrary to previous findings linking anemia to increased POD risk^[43]^. One possible explanation is that elevated hemoglobin levels may increase blood viscosity and impair microcirculation, thereby exacerbating cerebral hypoperfusion. However, this hypothesis requires further validation.

Correlation analyses indicated that N200 latency was positively correlated with surgery duration and negatively correlated with postoperative albumin on day 3, supporting the clinical relevance of these markers. No significant correlations were found between ERP latency and CRP or hemoglobin levels.

This study has several limitations. First, the small sample size may limit the generalizability of the findings and reduce statistical power. Second, only in-hospital data were analyzed; no long-term follow-up of cognitive outcomes was conducted. Third, the study lacked preoperative ERP data, making it difficult to assess individual baseline differences. Lastly, only male patients were included to control for gender-related variability in ERP signals^[44]^. Future studies should explore gender differences in ERP responses to POD and extend follow-up to assess long-term outcomes.

In summary, this study demonstrates that ERP components, particularly P300 and N200 latency, are sensitive to cognitive changes in patients with POD. ERPs may offer a valuable, non-invasive tool for early identification and risk stratification of POD in surgical patients. However, further large-scale, multicenter studies are needed to validate these findings and optimize their clinical application.

## Conclusion

Patients with POD demonstrated significant alterations in event-related potentials, particularly prolonged P300 and N200 latencies and a trend toward reduced amplitudes. These findings suggest that P300 and N200 may serve as promising, non-invasive electrophysiological markers for the detection of delirium; however, further studies with larger sample sizes and longitudinal designs are needed to validate their diagnostic utility.

## Data Availability

The data supporting the conclusion of this research have been included within the article. Datasets generated during and/or analyzed during the current study are publicly available.

## Research registration unique identifying number (UIN)

This study has been registered on https://www.clinicaltrials.gov (Clinical Trial Registration Number: NCT06082024).
